# Morphological and biochemical changes in the pancreas associated with acute systemic hypoxia

**DOI:** 10.1007/s13577-020-00481-0

**Published:** 2021-02-02

**Authors:** Fumiya Morioka, Naoto Tani, Tomoya Ikeda, Tatsuya Hirokawa, Kei Ikeda, Alissa Shida, Yayoi Aoki, Takaki Ishikawa

**Affiliations:** 1 Department of Legal Medicine, Osaka City University Medical School, 1-4-3 Asahi-machi, Abeno, Osaka, 545-8585 Japan; 2 Forensic Autopsy Section, Medico-Legal Consultation and Postmortem Investigation Support Center (MLCPI-SC), Osaka, Japan

**Keywords:** Hypoxia, Insulin, Glucose, Cortisol, Cell culture

## Abstract

This study aimed to investigate the changes associated with acute systemic hypoxia in the endocrine system, particularly in pancreatic tissues. The investigation was based on macroscopic, pathohistological, biochemical, and molecular biological findings in cell lines and human cadavers. The results showed that cases of death due to asphyxia more frequently showed severe subcapsular/interstitial hemorrhage versus the other causes of death. Histological examination showed that asphyxia cases were associated with severe morphological changes. Although measured insulin levels in the asphyxia were higher compared to other causes of death, no differences were noted for the glucagon and amylase levels with regard to the cause of death. Increased blood insulin levels were not associated with macro- and micromorphological changes, and did not show any association with glucose or cortisol levels. The experiment conducted under hypoxic conditions in cultured cells demonstrated that insulin mRNA expression and insulin protein levels peaked at 10 min after hypoxia exposure. However, there were no changes in either the amylase mRNA or protein levels. Corticosterone level peaked at 120 min after exposure to hypoxic conditions. Overall, acute systemic hypoxic conditions can directly affect the mechanisms involved in pancreatic insulin secretion.

## Introduction

Acute systemic hypoxia caused by asphyxia and acute circulatory failure due to cardiac disease substantially impacts various organs. Endocrine organs have an extensive vasculature making them particularly sensitive to systemic hypoxia [1–3]. Hypoxic conditions can have both morphological and functional impacts on the endocrine glands and hormone production [4, 5]. While studies have evaluated pathologies induced by acute systemic hypoxia, little research has addressed hypoxia’s effects on the endocrine system [6, 7].

As a result, we have conducted several studies designed to investigate endocrine system abnormalities that occur due to systemic hypoxia [8–10]. In an earlier report we found blood and cerebrospinal fluid (CSF) to have increased levels of triiodothyronine (T3) and thyroxine (T4). Thyroid gland secretions were high during acute hypoxic/ischemic conditions, regardless of any mechanical stimuli such as cervical compression [8]. We also found an increase in the pituitary gland blood-to-CSF transport of prolactin (PRL). This information led us to conclude that the pituitary gland receives neural stimulation under hypoxic conditions [9]. After looking at these results, we investigated exocrine and endocrine functions of the pancreas after acute hypoxia treatment and found very little literature. Our group went on to look at how the pancreas reacts to systemic hypoxic conditions. In this case we observed that subcapsular/interstitial hemorrhage occurs and serum amylase levels tend to be higher than the clinical reference range; which did not differ from levels seen in other causes of death [10]. This study investigated changes in the pancreas caused by acute systemic hypoxia with a particular focus on the effects on the endocrine cells in the human pancreas. We used cells from human autopsy cases where the body experienced acute systemic hypoxia as the cause of death. We also used cultured cells to conduct experiments during hypoxic conditions to further investigate the autopsy results.

## Materials and methods

### Autopsy materials

We examined a total of 94 serial forensic autopsy cases (66 males and 28 females: median age at death was 62 years) within 72 h postmortem. The inclusion criteria were cases where witnesses provided well-established circumstantial evidence confirming the injury/cause of death, and where the time of death was well defined [11]. A complete autopsy which included macromorphological, micropathological, toxicological, and postmortem radiographic examinations identified the following causes of death: asphyxia (systemic hypoxia disorder), sharp instrument injury (hemorrhagic shock), blunt injury (head injury and non-head injury), fire fatality (burn and carbon monoxide intoxication), drowning (alveolus injury), and acute cardiac death (cardiac failure) (Table 1). Patients with diabetes mellitus and liver cirrhosis were excluded from this study, as well as subjects with hyperthermia (heat stroke), crush syndrome, and intoxication due to the effect hemolysis may have on insulin concentrations [12, 13].

**Table 1:** Case profile

Cause of death	*n*	Male/female	Age (y) (median)	Emergency medical care	Survival period (h)	Postmortem period (h) (median)
Asphyxia	22	14/8	6–87 (50.5)	0	<0.5	6–60 (30)
Blunt injury	15	12/3	30–84 (57)	0	<0.5	6–48 (24)
Head injury	6	5/1	32–83 (55.5)	0	<0.5	6–36 (19)
Non-head injury	9	7/2	30–84 (57)	0	<0.5	12–48 (20)
Sharp instrument injury	11	9/2	22–75 (57)	0	<0.5	6–60 (18)
Fire fatality	23	16/7	33–91 (77)	0	<0.5	6–60 (29)
CO-Hb < 30%	12	8/4	33–91 (75.5)	0	<0.5	6–60 (31)
CO-Hb 30–60%	6	5/1	41–85 (81)	0	<0.5	12–48 (24)
CO-Hb > 60%	5	3/2	43–87 (65)	0	<0.5	24–60 (25)
Drowning	11	3/8	30–96 (65)	0	<0.5	6–60 (31)
Acute cardiac death	12	12/0	16–83 (63)	0	<0.5	12–60 (30)
Total	94	66/28	6–96 (62)	0	<0.5	6–60 (30)

*CO-Hb* carbon monoxide hemoglobin

### Macropathological findings

During the autopsy we determined pancreatic subcapsular/interstitial hemorrhage frequency in accordance with the cause of death. Macroscopic pancreatic subcapsular/interstitial hemorrhage was scored according to the range and degree of bleeding as follows: 0, no hemorrhage; 1, local (slight) hemorrhage; 2, moderate hemorrhage; 3, diffuse (severe) hemorrhage.

### Micropathological and immunohistochemical analyses

Serial pancreatic specimens were used for hematoxylin–eosin staining. Scoring of the microscopic pancreatic tissue pattern changes across five random pancreatic tissues were based on three degrees of severity that included: none-mild type, scattering type, and diffuse type.

Pancreas hemorrhagic samples due to sharp instrument injury were collected and maintained at room temperature (postmortem period of about 24 h) and then compared with the subsequent postmortem morphological changes. Histopathological findings were confirmed for each of the observation points (0 h, 12 h, 24 h, 2 days, 3 days, 4 days), respectively.

For the immunohistochemical analyses, the specimen was incubated overnight with insulin rabbit polyclonal antibodies (15848-1-AP; Proteintech, Rosemont, IL, USA) [14, 15] and glucagon (15954-1-AP; Proteintech) [15, 16] at room temperature. The total number of cells in the islets of Langerhans was counted for each specimen under 200 × magnification, as well as the number of insulin- and glucagon-positive cells in comparison to the total number of Langerhans cells.

### Biochemical analyses

#### Measurement of blood insulin, glucagon, and amylase concentrations

Serum insulin concentrations were measured by a chemiluminescent enzyme immunoassay [17–19] using a fully automated chemiluminescent enzyme immunity measurement system (Lumipulse L2400: Fujirebio Inc., Tokyo, Japan). After aseptically collecting blood samples from the right heart chambers, all samples were immediately centrifuged to separate the sera. All measurements used the clinical serum insulin reference range 1.84–12.2 µIU/mL [20].

Serum glucagon concentrations were measured by radioimmunoassay (RIA) [21, 22] using a gamma counter (ARC-8010, Hitachi, Ltd., Tokyo, Japan) and the Glucagon RIA “SML” (Euro-Diagnostica AB, Denis Pharma K.K., Tokyo, Japan), a glucagon measurement kit for plasma. This kit is based on the radioimmunoassay principle that uses a double antibody technique for determining the bound/free separation. For this measurement we used the clinical serum glucagon reference range of 70–174 pg/mL [23].

In some cases, serum amylase concentrations were spectrophotometrically measured (JCA-BM8000: JEOL Inc., Tokyo, Japan) [24]. For this measurement, the clinical serum amylase reference range used was 37–125 U/L.

#### Method and apparatus for measuring blood glucose and cortisol concentrations

Blood glucose was measured using the hexokinase UV method [25–27]. Measurements were carried out using an automatic analysis device (JCA-BM6070, JEOL, Tokyo, Japan) and SHIKA liquids GLU J (Kanto Kagaku, Tokyo, Japan) as the reagent. The sample consisted of 0.5 mL of serum from the right heart using a standard measurement method with a clinical serum glucose concentration reference range of 70–109 mg/dL [28, 29].

Right heart serum cortisol levels were measured using a competitive fluorescent enzyme immunoassay on the AIA-3601 analyzer (Tosoh Bioscience GmbH, Griesheim, Germany) [30, 31]. The lower and upper reported values for the cortisol assays were 0.2 and 60 µg/dL, respectively.

### Cell culture under hypoxic conditions

#### Insulin- and amylase-secreting cell line

This study used the BRIN-BD11 cell line, an insulin-secreting hybrid cell line formed by electrofusion between a primary culture of NEDH rat pancreatic islets and RINm5F (a cell line derived from a NEDH rat insulinoma). BRIN-BD11 has been shown to be tumorigenic when transplanted into SCID mouse hosts. Moreover, this cell line can be used for the study of pancreatic beta cell function [32]. The culture medium consisted of RPMI1640 supplemented with 10% fetal bovine serum (FBS). Cells were cultured in a humidified atmosphere of 4.7% CO_2_ and 5% O_2_ at 37 °C. The cell count was measured using the Cell Counter model R1 (Olympus Optical Co., Ltd., Tokyo, Japan) following a trypan blue-exclusion test and then adjusted to 1.4 × 10^6^ cells/mL until 75 min of culture. Culture fluid insulin levels were subsequently measured via an enzyme-linked immunosorbent assay using a rat insulin measurement kit (Morinaga Institute of Biological Science Inc., Kanagawa, Japan). We also measured mRNA levels using reverse transcription-polymerase chain reaction (RT-PCR) after hypoxic exposure.

We added actinomycin D (2 μg/mL) to the BRIN-BD11 cells to block reproduction and transcription from DNA. Then we measured insulin, HIF1α, and vascular endothelial growth factor (VEGF) with and without actinomycin D.

AR42J cells are derived from a rat pancreatic external secretion adenocarcinoma [33]. We cultured the AR42J cells in a humidified atmosphere of 4.7% CO_2_ and 5% O_2_ at 37 °C. Cell counts were done using the Cell Counter model R1 (Olympus Optical Co., Ltd., Tokyo, Japan), with the cells adjusted to 1.0 × 10^6^ cells/mL until 24 h of culture. Culture fluid amylase levels were also measured using a colorimetric analysis (SPOTCHEM D-Concept^™^, ARKRAY Inc., Kyoto, JAPAN) [34].

#### Corticosterone-secreting Y-1 adrenal cell line

Corticosterone-secreting Y-1 adrenal cells derived from mice were developed to verify whether these cells secrete hormones upon stimulation due to exposure to hypoxia. Mouse Y-1 adrenocortical tumor cells (ATCC, CCL-79) were established in a male mouse [35, 36]. The culture medium consisted of a 1:1 ratio of DMEM-F12 and 15% charcoal stripped FBS (Biological Industries, Cromwell, CT, USA) with 4 mM L-glutamine, 50 U/mL penicillin and 50 μg/mL streptomycin. Initially, cells of both types were seeded and cultured at 37 °C. Growth was controlled at about 1 × 10^5^ cells/cm^2^ for Y-1, with the cells allowed to proliferate until they covered the surface of the culture dish. After the Y-1 cells reached confluence, they were then transferred to 1% O_2_ and maintained. The amount of corticosterone in the culture medium was measured at 10, 30, 60, 120, 240, 480, 720, 1440 (1 day), 2880 (2 days), and 4320 min (3 days). Corticosterone was measured using a mouse corticosterone assay kit (ELISA kit, No: #501320, Cayman Chemical Company, Ann Arbor, MI, USA) [37, 38]. At the end of the experiment, adherent cells were dissociated from the surface using trypsin and then counted, hormone concentrations were then calculated using a correction formula in conjunction with the measured values. We measured HIF1α (Sandwich ELISA kit, CBA-280, Cell Biolabs Inc., San Diego, CA, USA) [39, 40] and VEGF (ELISA kit, ab209882, Abcam, Tokyo, Japan) as the control [41].

### Quantitative reverse transcription-polymerase chain reaction of cultured cells

Total RNA was isolated using Isogen reagent (Nippon Gene, Toyama, Japan) in accordance with the manufacturer’s instructions. cDNA copies of the total RNA were synthesized using the High-Capacity RNA-to-cDNA Kit (Applied Biosystems, Foster City, CA, USA). The reaction mixture included 9 µL samples of total RNA, 10.0 µL of 2 × RT buffer, and 1.0 µL of 20 × RT enzyme mix. Conditions for the reverse transcription were as follows: 37 °C for 60 min and then 95 °C for 5 min. A total of 20.0 µL of reaction mixture containing 10.0 µL of TaqMan gene expression master mix (2 ×), 1.0 µL of TaqMan gene expression assay (20 ×), 4 µL of cDNA, and 5 µL of H_2_O were added to each well of a Fast 96-Well Reaction Plate (0.1 mL, Applied Biosystems). Quantitative RT-PCR was performed using primers for Insulin 1 (Ins1: TaqMan assay ID: Rn0212433_g1), Insulin 2 (Ins2: TaqMan assay ID: Rn01774648_g1), and amylase (Amy 2a3: TaqMan assay ID: Rn00821330-g1) on a StepOnePlus real-time PCR system (Applied Biosystems). The threshold cycle was calculated automatically using the instrument software, with a threshold value of 0.2. For this study, β-actin (TaqMan assay ID: Rn00667869_m1) was used as the endogenous reference gene. TaqMan Gene Expression Assays were purchased from Applied Biosystems and tested in accordance with the manufacturer’s protocol [42].

### Structural imaging using a transmission electron microscope

Cultured endocrine cells (BRIN-BD11) and acinar cells (AR42J) of the pancreas were collected under conditions of hypoxia. The cells were prefixed using 2.5% glutaraldehyde and 2% paraformaldehyde buffered with 0.1 M phosphate buffer (pH 7.4) at 4 °C overnight and then fixed with 1% osmium tetroxide buffered with 0.1 M phosphate buffer for 2.5 h at 4 °C. Ultrathin sections were prepared from resin blocks using a diamond knife on an ultramicrotome (Ultracut UCT, Leica, Vienna, Austria). Sections were stained with 5% uranyl acetate in 50% ethanol for 20 min and Reynolds’ lead citrate for 3 min. Stained sections were then analyzed using TEM (H-7500, Hitachi) [43].

### Statistical analyses

We used Fisher’s exact test to compare the parameters of pancreatic macromorphological findings between the causes of death. *p* values for the tests were adjusted in accordance with the Benjamini–Hochberg false discovery rate method. The same methodology was used to compare the micro pancreatic tissue findings. Spearman’s rank correlation coefficient was used to examine the correlation between the two continuous parameters. The Steel–Dwass test was used for multiple comparisons among the groups. In addition, the utility of the serum insulin and glucagon levels for differentiating between death due to asphyxia and other causes was evaluated using receiver operating characteristic (ROC) curves and the areas under the curves. Youden’s index (sensitivity + specificity − 1) was used to determine the best cut-off values. Results are presented as medians and interquartile ranges, unless otherwise stated. A *p* value of < 0.05 was considered significant. All statistical procedures, including the ROC analyses, were performed using SPSS 9.0 (SPSS Inc., Chicago, IL, USA).

## Results

### Macroscopic pancreatic subcapsular/interstitial hemorrhage ratio and histopathological pancreatic tissue pattern changes associated with the cause of death

**Fig. 1:**
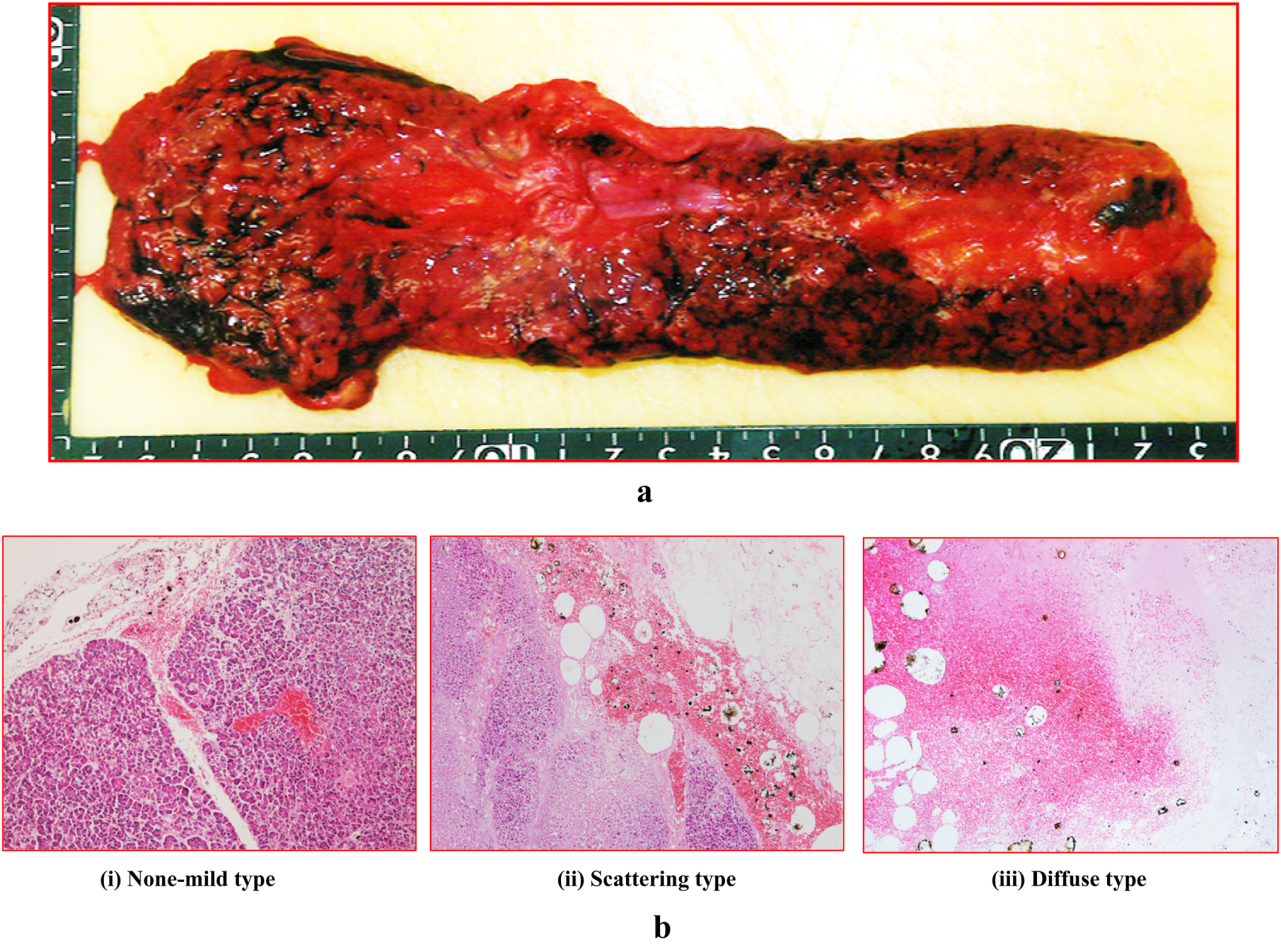
**a** Macromorphological findings showing pancreatic subcapsular/interstitial hemorrhage without parenchymal injury. **b** Panels (i)–(iii) show each of the histological findings (a: none to mild type, b: scattering type, c: diffuse type). Figure indicates (i) male in his 50 s, asphyxia, postmortem period 18 h; (ii) female in her 80 s, fire fatality, postmortem period 30 h; (iii) male in his 30 s, asphyxia, postmortem period 20 h

Macromorphological findings indicated that there were pancreatic subcapsular/interstitial hemorrhages present without any observed injury (Fig. 1a). Histopathological findings showed there were three patterns of pancreatic tissue damage, which included both the exocrine and endocrine glands. Figure 1b shows each of the histological findings for the asphyxia cases [(i) none to mild type, (ii) scattering type, (iii) diffuse type].

**Fig. 2:**
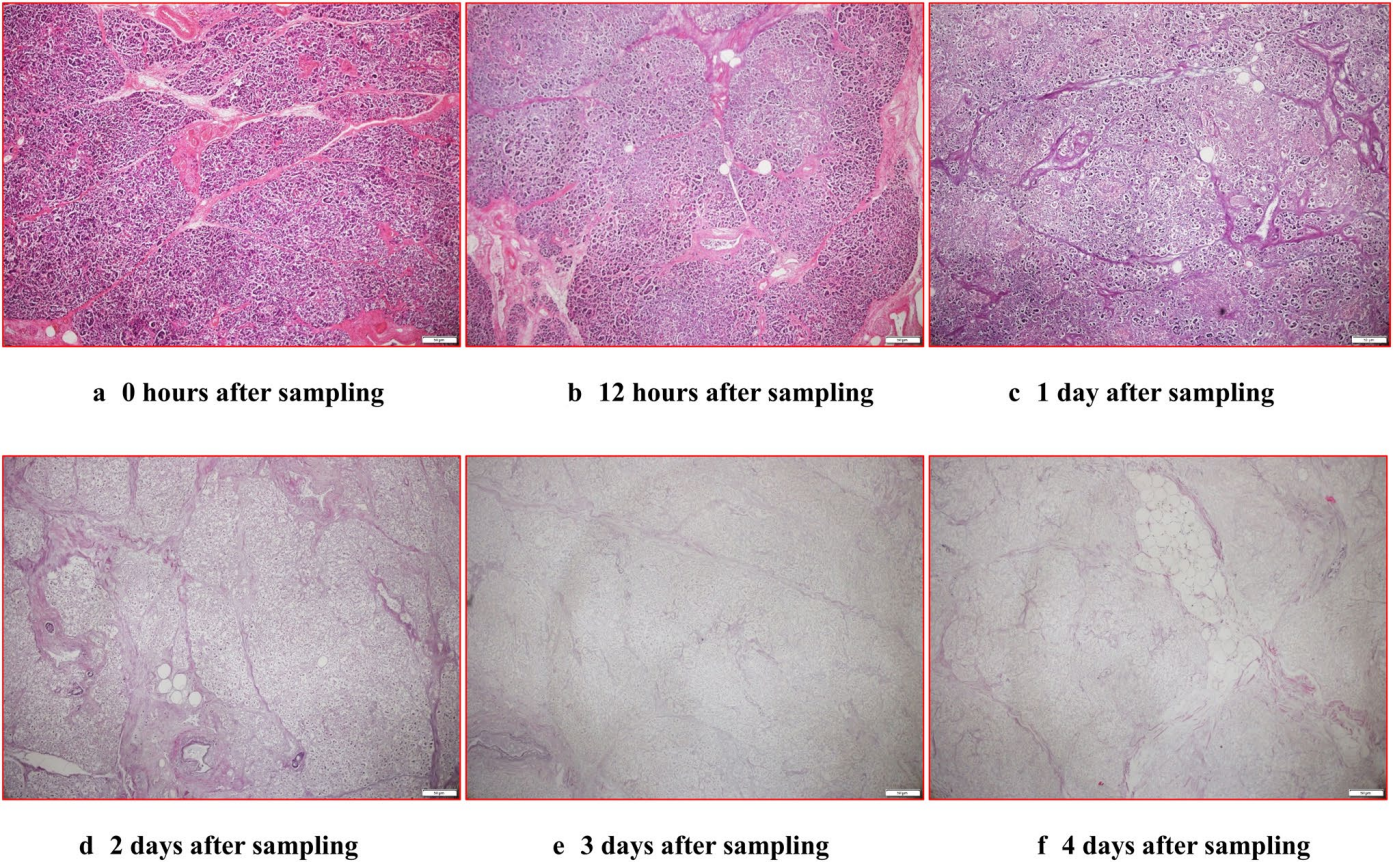
The figure shows the changes observed for the elapsed times for each of the extracted pancreas samples at autopsy. **a** 0 h after sampling, **b** 12 h after sampling, **c** 1 day after sampling, **d** 2 days after sampling, **e** 3 days after sampling, **f** 4 days after sampling

**Fig. 3:**
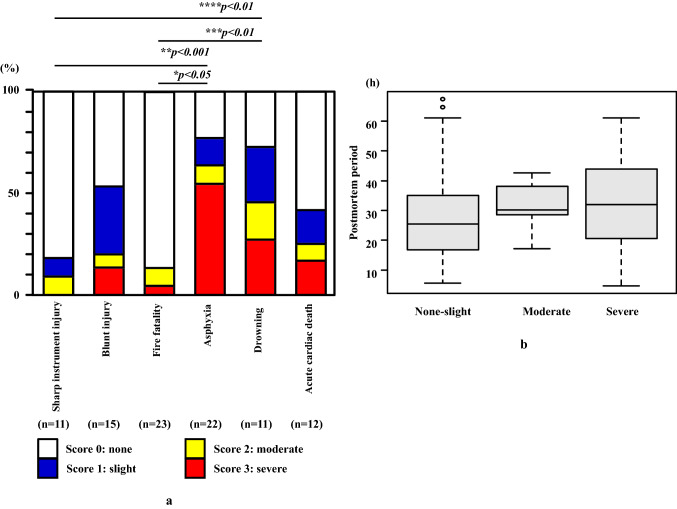
**a** Range and/or degree of macroscopic pancreatic subcapsular/interstitial hemorrhage according to the cause of death. Moderate to high scores (score of 2 and 3) were observed in the asphyxia cases. **b** Graph showing a ratio of macroscopic pancreatic tissue hemorrhage levels in the postmortem period. There was no significant relationship observed between the postmortem period and pancreatic hemorrhage degree (*p* > 0.05)

In contrast, when changes in the pancreatic tissue based on the postmortem period were evaluated, there were no differences observed after 12 h. The micromorphological changes appeared conspicuously after 2 days of the postmortem period, with pancreatic cells diffusely falling off. Two days posthumously, the histologic form was no longer maintained (Fig. 2a–f). Individuals who died due to asphyxia (77.3%) had a higher incidence of macroscopic pancreatic subcapsular/interstitial hemorrhage as compared to those who died due to other causes (13.0–53.3%, mean 27.9%) with the exception for drowning (72.7%) (Fig. 3a and Table 2). Those who died due to asphyxia had macroscopic pancreatic subcapsular/interstitial hemorrhage score classified as severe (score of 3) (Fig. 3a and Table 2). There was no relationship found between the macroscopic pancreatic subcapsular/interstitial hemorrhage score and the postmortem period (Fig. 3b). Statistical evaluation demonstrated that the moderate or severe hemorrhage cases were higher for asphyxia as compared to sharp instrument injury and fire fatality (*p* < 0.05*, p* < 0.001) (Fig. 3a).

**Table 2:** Results of the macromorphological, micropathological, and immunohistochemical examinations in the pancreas

Cause of death	Number (*n*)	Macromorphological interstitial/subcapsular hemorrhage (None–slight/moderate/severe) (Number: *n*)	Micropathological tissue changes (None–mild/scattering/diffuse) (Number: *n*)	Immunohistochemical staining	
				Insulin (%; median)	Glucagon (%; median)
Asphyxia	22	8/2/12	2/5/15	41.6–80.8 (61.4)	15.5–38.6 (28.3)
Blunt injury	15	12/1/2	6/3/6	44.7–69.5 (60.7)	14.7–39.4 (27.1)
Head injury	6	5/1/0	2/1/3	44.7–65.3 (60.8)	22.8–35.7 (30.1)
Non-head injury	9	7/0/2	4/2/3	49.9–69.5 (59.4)	14.7–39.4 (25.6)
Sharp instrument injury	11	10/1/0	5/4/2	34.7–77.4 (58.8)	17.8–46.7 (32.1)
Fire fatality	23	20/2/1	11/6/6	39.0–70.9 (55.1)	13.9–70.5 (26.2)
CO-Hb < 30%	12	11/1/0	5/5/2	39.0–70.5 (53.8)	15.0–70.5 (25.7)
CO-Hb 30–60%	6	5/1/0	2/1/3	48.2–70.9 (60.6)	13.9–32.9 (23.2)
CO-Hb > 60%	5	4/0/1	4/0/1	43.5–62.4 (54.6)	22.0–36.6 (27.7)
Drowning	11	6/2/3	6/2/3	45.8–70.6 (59.5)	17.4–48.2 (30.6)
Acute cardiac death	12	9/1/2	1/5/6	38.6–74.7 (63.4)	15.6–54.3 (35.3)
Total	94	65/9/20	31/25/38	34.7–80.8 (59.4)	13.9–70.5 (28.9)

*CO-Hb* carbon monoxide hemoglobin

**Fig. 4:**
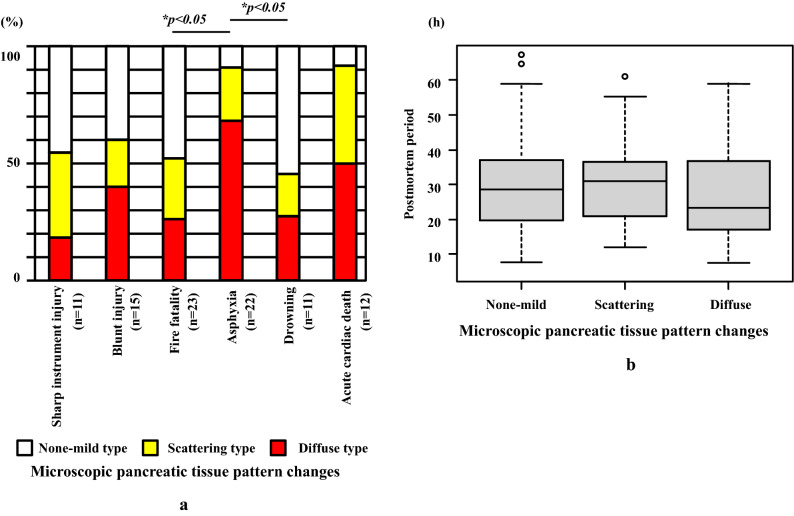
**a** Graph showing score ratios for microscopic pancreatic tissue pattern changes. Asphyxia cases exhibited a scattering and diffuse pattern (moderate to severe) as compared to the other causes of death. **b** Graph showing the ratio of the microscopic pancreatic tissue pattern changes in the postmortem period. There was no significant relationship between the postmortem period and microscopic pancreatic tissue pattern changes (*p* > 0.05)

Histopathological analyses demonstrated that diffuse type of pancreatic tissue damage changes, including acinar and islet cells, were found more often for asphyxia (90.9%) as compared to those who died from other causes (45.5–60.0%, mean 53.3%), excluding acute cardiac death (Fig. 4a and Table 2). No significant differences were noted between the pancreatic micromorphological tissue changes and the postmortem period (Fig. 4b).

### Immunohistochemical analysis

**Fig. 5:**
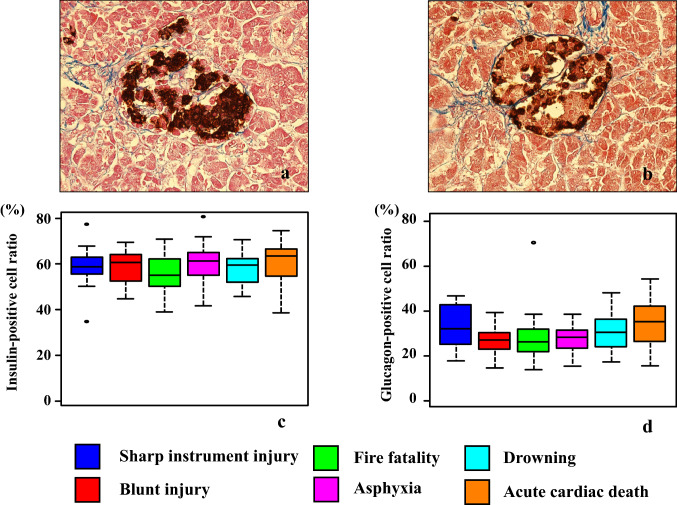
The figure shows the insulin (**a**) and glucagon (**b**) immunostaining in the Langerhans cells of the pancreas. Immunohistochemical findings for the percentage of insulin-positive (**c**) and glucagon-positive (**d**) cells in the Langerhans cells. There were no significant differences for the percentage of insulin- or glucagon-positive cells found among all causes of death (*p* > 0.05)

The islets of Langerhans exhibited staining for both insulin and glucagon (Fig. 5a, b). There were no significant differences in the percentages of the insulin- and glucagon-positive cells observed among the causes of death in the islets of Langerhans (Fig. 5c, d, and Table 2).

### Postmortem serum insulin, glucagon, and amylase levels

**Fig. 6:**
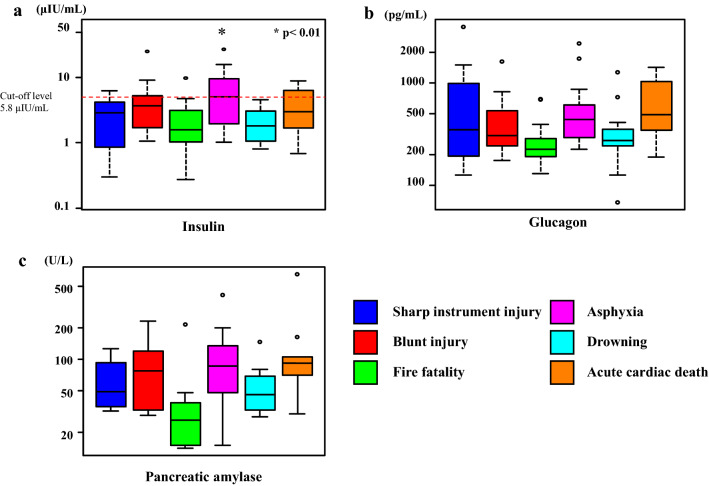
Insulin (**a**), glucagon (**b**), and amylase (**c**) levels in the right heart blood according to the cause of death in the autopsy cases. Asphyxia cases show significantly higher blood insulin levels as compared to the other causes of death (*p < 0.01). No significant differences were observed for the blood glucagon and amylase levels in accordance with the cause of death (*p* > 0.05)

Serum insulin levels were slightly negatively correlated with the subject’s age (*r* =  − 0.324, *p* < 0.01), but not with sex or the postmortem period. Those who died due to asphyxia had higher serum insulin levels (median, 5.0 µIU/mL) as compared to those who died due to other causes (median, 2.1 µIU/mL). A cut-off insulin level of 5.8 µIU/mL (sensitivity 0.861; specificity 0.500) was identified for predicting asphyxia (Fig. 6a and Table 3).

**Table 3:** Blood insulin, glucagon, amylase, cortisol, and glucose levels in the right heart blood

Cause of death	Number (*n*)	Right heart blood				
		Insulin (μIU/mL) (median)	Glucagon (pg/mL) (median)	Amylase (U/L) (median)	Cortisol (μg/dL) (median)	Glucose (mg/dL) (median)
Asphyxia	22	1.0–26.4 (5.0)	224–2445 (440)	15–412 (86)	3.3–24.6 (9.6)	7–830 (302.5)
Blunt injury	15	1.1–24.4 (3.7)	175–1630 (305)	29–231 (77)	2.7–61.2 (13.8)	3–502 (167)
Head injury	6	1.1–24.4 (3.5)	213–820 (371)	58–231 (120)	2.7–61.2 (9.9)	3–185 (97)
Non-head injury	9	1.3–8.9 (4.3)	175–1630 (305)	29–135 (34)	3.4–31.8 (13.9)	50–502 (325)
Sharp instrument injury	11	0.3–6.2 (2.9)	126–3550 (350)	32–126 (49)	0.2–19.6 (10.3)	1–440 (90)
Fire fatality	23	0.3–10.5 (1.7)	130–700 (226)	14–216 (26)	2.3–39.0 (13.1)	4–708 (121)
CO-Hb < 30%	12	0.3–5.1 (1.5)	130–700 (239.5)	15–37 (19)	3.0–39.0 (14.5)	4–441 (131)
CO-Hb 30–60%	6	1.1–3.9 (1.8)	178–358 (201)	14–216 (15)	2.3–20.6 (9.6)	32–708 (228.5)
CO-Hb > 60%	5	0.3–10.5 (3.1)	182–287 (229)	15–48 (39)	10.2–27.3 (23.8)	34–680 (110)
Drowning	11	0.8–4.5 (1.8)	68–1270 (275)	28–147 (45)	3.4–33.0 (14.4)	1–693 (273)
Acute cardiac death	12	0.7–8.7 (2.9)	188–1418 (492.5)	30–654 (92)	4.8–72.5 (10.9)	1–546 (248.5)
Total	94	0.3–26.4 (2.8)	68–3550 (299.5)	14–654 (43)	0.2–72.5 (12.7)	1–830 (217.5)

*CO-Hb* carbon monoxide hemoglobin

Serum glucagon levels were found to have no relationship with the age, sex, and postmortem period. For all causes of death, the glucagon level was higher than the upper clinical reference range. However, among all causes of death, there was no significant difference noted in the serum glucagon levels (Fig. 6b and Table 3).

There was also no relationship found between the serum amylase levels and the age, sex, and postmortem period. For all causes of death, amylase levels were approximately within the clinical reference range. There were no groups with conspicuously high amylase levels (Fig. 6c and Table 3).

**Fig. 7:**
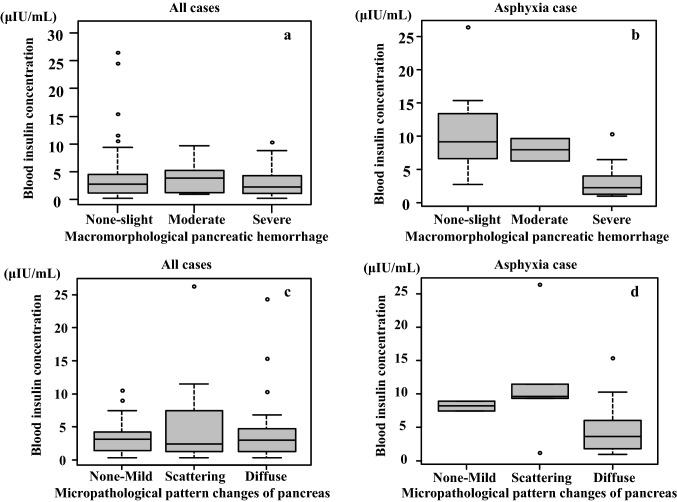
Comparison between macromorphological pancreatic hemorrhage levels and blood insulin levels in cases with pancreatic subcapsular/interstitial hemorrhage. There was no relationship observed between the hemorrhage levels and blood insulin levels (a: all cases, b: asphyxia cases) (*p* > 0.05). Histological comparison between the pattern changes in the tissue and blood insulin level in the pancreas. There was no relationship observed between the tissue pattern changes and the blood insulin level in the cases (c: all cases, d: asphyxia cases) (*p* > *0.05)*

The macromorphological hemorrhage level and micropathological pattern changes were found to have no relationship with either the serum insulin or glucagon levels (Fig. 7a–d). The macromorphological severe hemorrhage cases tended to exhibit low serum insulin levels even though there was no statistic significant difference observed when the asphyxia cases were compared (Fig. 7b).

### Postmortem serum cortisol and glucose levels

**Fig. 8:**
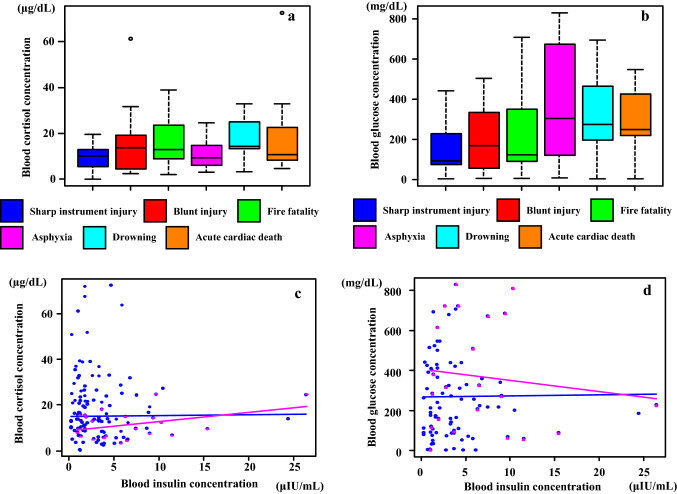
Blood cortisol (**a**) and glucose (**b**) concentrations according to the cause of death in autopsy cases. There was no relationship observed between the cortisol concentrations and causes of death (*p* > 0.05). Evaluation of the correlation between the blood cortisol (**c**), glucose (**d**), and the blood insulin concentrations revealed that there was no relationship between these groups [blue lines and circles: all cases (*p* > 0.05); red lines and triangles: asphyxia cases (*p* > 0.05)]

There were no significant differences observed in the serum cortisol levels among the causes of death (Fig. 8a and Table 3). While the glucose level tended to be high for asphyxia (7.0–830.0 mg/dL, median 302.5 mg/dL), drowning (1.0–693.0 mg/dL, median 273.0 mg/dL), and acute cardiac death (1.0–546.0 mg/dL, median 248.5 mg/dL), there was no statistically significant difference noted for all causes of death (1.0–830.0 mg/dL, median 217.5 mg/dL) (Fig. 8b and Table 3).

The serum insulin levels in the present study did not exhibit any correlation with serum cortisol or glucose (Fig. 8c, d).

### Insulin concentrations and insulin mRNA levels in the conditioned medium BRIN-BD11 cultured under hypoxic conditions

**Fig. 9:**
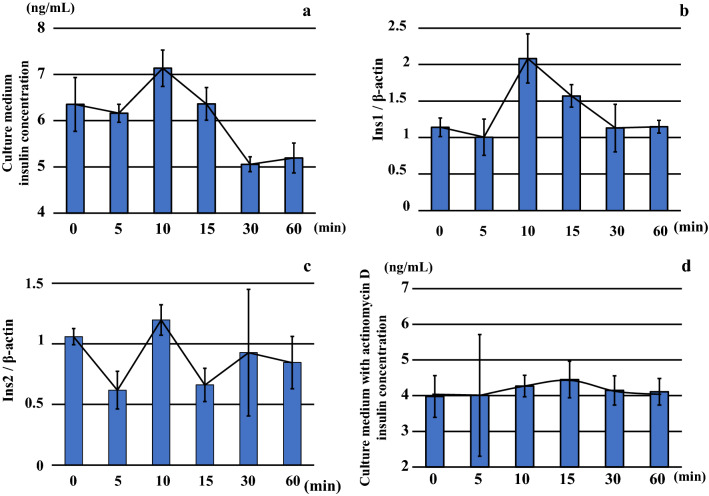
Insulin concentrations (**a**) and insulin mRNA levels, including Ins1 (**b**) and Ins2 (**c**), in the conditioned medium BRIN-BD11 cultured under hypoxic conditions (O_2_ 5%). Insulin levels were highest after 10 min of incubation under hypoxic conditions. However, insulin levels gradually decreased after 15 min. With regard to insulin mRNA expressions, Ins1 expression increased after incubation for 10 min under hypoxic conditions. Ins2 expression did not exhibit any remarkable change under the hypoxic conditions as compared to that observed for Ins1 mRNA expression. Furthermore, after adding actinomycin D to the BRIN-BD11 cells, there was inhibition of the reproduction and transcription from the DNA. Results showed that the insulin exhibited low levels as compared to the actinomycin D non-addition experiment (**d**)

BRIN-BD11 cells showed increased insulin levels following incubation for 10 min under 5% hypoxic conditions (Fig. 9a and Table 4). However, the insulin levels decreased gradually after 15 min.

**Table 4:** Insulin, HIF1α, and VEGF in BRIN-BD11 with and without actinomycin D, and mRNA insulin level in BRIN-BD11

Cultured time under hypoxia (min)	Pancreatic endocrine cell culture (BRIN-BD11)					Pancreatic endocrine cell culture (BRIN-BD11) with actinomycin D		
	Insulin (ng/mL)	Ins1/β-actin	Ins2/β-actin	HIF1α (ng/mL) (median)	VEGF (ng/mL) (median)	Insulin (ng/mL) (median)	HIF1α (ng/mL) (median)	VEGF (pg/mL) (median)
0	5.58–6.99 (6.49)	1.00–1.31 (1.11)	1.00–1.15 (1.02)	0.48–3.10 (1.74)	1017.0–1250.0 (1209)	3.12–4.56 (4.35)	1.07–5.11 (3.20)	367.4–485.9 (439.2)
5	5.89–6.36 (6.23)	0.72–1.33 (0.97)	0.50–0.84 (0.52)	0.52–4.19 (2.32)	957.7–1190.0 (1014)	2.40–7.10 (3.12)	0.68–5.68 (3.08)	368.2–484.3 (428.1)
10	6.58–7.46 (7.36)	1.78–2.55 (1.92)	1.02–1.30 (1.27)	0.59–6.59 (1.92)	950.0–1159.0 (1131)	3.70–4.58 (4.33)	2.46–5.37 (2.63)	396.8–478.9 (432.5)
15	5.89–6.73 (6.47)	1.39–1.77 (1.55)	0.47–0.76 (0.75)	0.45–5.66 (2.92)	1247.0–1694.0 (1336)	3.97–5.43 (4.22)	0.83–8.54 (2.59)	391.1–552.0 (413.9)
30	4.86–5.25 (5.06)	0.89–1.59 (0.91)	0.31–1.59 (0.88)	2.97–7.33 (4.39)	1138.0–1684.0 (1401)	3.50–4.72 (4.22)	1.45–5.42 (2.74)	391.9–540.7 (438.4)
60	4.73–5.45 (5.39)	1.06–1.27 (1.12)	0.55–1.07 (0.92)	1.19–4.46 (2.47)	1062.0–1331.0 (1268)	3.69–4.72 (4.07)	0.65–7.63 (4.32)	376.7–501.4 (449.8)

Evaluation of the insulin mRNA expression demonstrated that the Ins1 expression increased after incubation for 10 min under hypoxic conditions in conjunction with the insulin levels in the culture medium (Fig. 9b and Table 4). Ins2 expression exhibited a high level within 10 min, but the levels and variations were both low as compared to that observed for Ins1 under hypoxic conditions (Fig. 9c and Table 4).

After adding actinomycin D to the BRIN-BD11 cells, we measured the insulin concentrations. Results showed that the insulin concentrations were all at low levels including the actinomycin D in comparison with excluding the actinomycin D (Fig. 9d and Table 4).

### HIF1α and VEGF concentrations in the conditioned medium BRIN-BD11 when cultured under hypoxic conditions

**Fig. 10:**
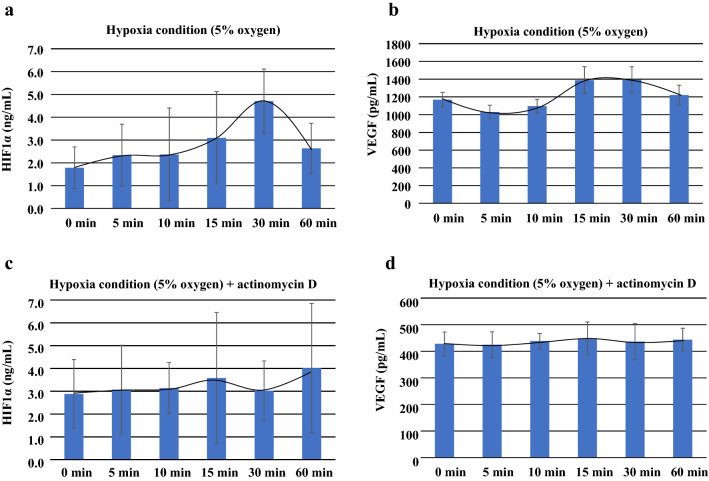
Graph shows the HIF1α and VEGF concentrations in the conditioned medium BRIN-BD11 that was cultured under hypoxic conditions. **a** Levels of HIF1α and VEGF, which were used as the hypoxia control markers for cellular function during the hypoxic conditions in the BRIN-BD11 cells, increased starting from 30 min in HIF1α. **b** The graph shows the VEGF concentrations from 15 min to 60 min, with the maximum reached from 15 min to 30 min. The figure also shows the HIF1α (**c**) and VEGF (**d**) levels after the addition of actinomycin D during the conditions of hypoxia. The addition of actinomycin D in the cultured cells during the hypoxia, led to smaller changes in both the HIF1α and VEGF

HIF1α and VEGF were used as control markers for cellular function during hypoxic conditions in BRIN-BD11 cells. HIF1α levels increased and reached maximum secretion at 30 min (2.97–7.33 ng/mL, median 4.39 ng/mL) (Fig. 10a and Table 4). VEGF levels increased from 15 min (1247.0–1694.0 pg/mL, median 1336 pg/mL) until 30 min (1138.0–1684.0 pg/mL, median 1401 pg/mL), with the maximum reached at 60 min (Fig. 10b and Table 4).

After the addition of actinomycin D during hypoxia, the HIF1α (Fig. 10c and Table 4) and VEFG (Fig. 10d and Table 4) levels exhibited a smaller and less significant difference for the measurements taken after initial administration (Table 4).

### Amylase concentrations and amylase mRNA levels in the conditioned medium AR42J cultured under hypoxic conditions

**Fig. 11:**
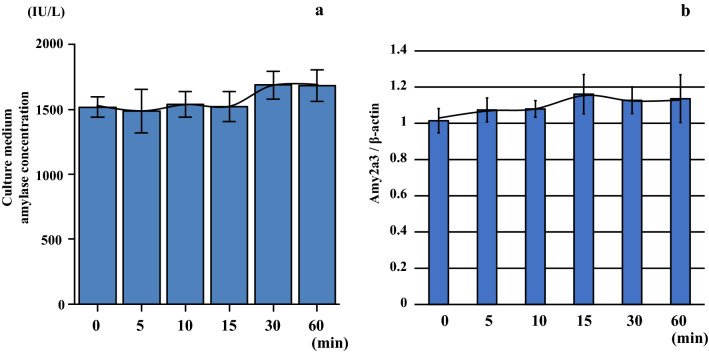
AR42J cells exhibited no increased amylase levels (**a**) or amylase mRNA (Amy2a3) levels (**b**) in the culture medium following incubation for 0–60 min under hypoxic conditions (O_2_ 5%) at each of the respective time points

The AR42J cells exhibited no increase in amylase levels following a 0–60 min incubation under 5% hypoxic conditions in the conditioned medium (Fig. 11a and Table 5), and the mRNA amylase level was similar to the amylase concentration at each of the time points (Fig. 11b and Table 5).

**Table 5:** Amylase concentrations and mRNA levels in the conditioned medium AR42J cultured under hypoxic conditions

Cultured time under hypoxia (min)	Pancreatic acinar cell culture (AR42J)	
	Amylase (IU/L) (median)	Amy2a3/β-actin
0	1431–1635 (1516.5)	1.00/1.13/1.01/1.02/0.90/1.00
5	1200–1692 (1491.0)	1.01/1.01/1.00/1.08/1.17/1.14
10	1407–1677 (1552.5)	1.07/1.03/1.15/1.01/1.07/1.11
15	1422–1752 (1488.0)	1.09/1.02/1.18/1.13/1.14/1.37
30	1578–1887 (1656.0)	1.15/1.13/1.05/1.25/1.03/1.12
60	1563–1902 (1674.0)	1.03/1.20/1.16/1.16/1.33/0.91

### Transmission electron microscope imaging in the BRIN-BD11 and AR42J cell culture model

**Fig. 12:**
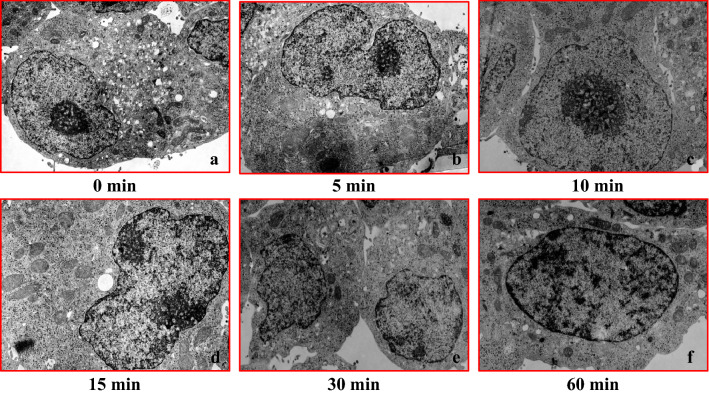
Transmission electron microscopy images of the BRIN-BD (rat islets) cell culture model during conditions of hypoxia (O_2_ 5%). Cell structure remained normal until approximately 10 min after the hypoxia. However, mitochondrial swelling and nuclear structure collapse findings became apparent around 15 min after the hypoxia (hypoxic condition period: **a** 0 min, **b** 5 min, **c** 10 min, **d** 15 min, **e** 30 min, and **f** 60 min)

After culturing pancreatic internal secretion cells (BRIN-BD11) under hypoxia, changes at each time point were evaluated using an electron microscope. Results showed that the cell structure was maintained for approximately 10 min under conditions of hypoxia. However, mitochondrial swelling and nuclear structure collapse started to become apparent after approximately 15 min under hypoxia (Fig. 12a–f).

**Fig. 13:**
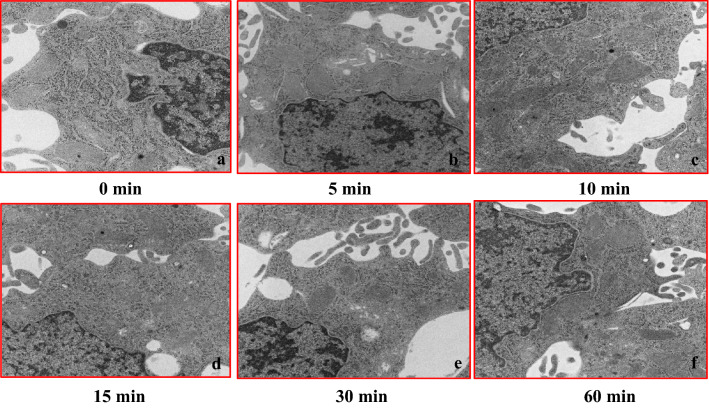
Transmission electron microscopy images of the AR42J (rat acinar cells) cell culture model during conditions of hypoxia (O_2_ 5%). In the AR42J cells, there were no changes in the cell organelles as the hypoxia progressed as compared to the pancreatic internal secretion cells (BRIN-BD11), with the exception for mitochondrial edema (**a**–**f**)

There were no changes in the cell organelles between the pancreatic external secretion cell line (AR42J) and the BRIN-BD11 cell line, with the exception of mitochondrial edema (Fig. 13a–f).

### Corticosterone, HIF1α, and VEGF concentrations in Y-1 cells cultured under hypoxic conditions

**Fig. 14:**
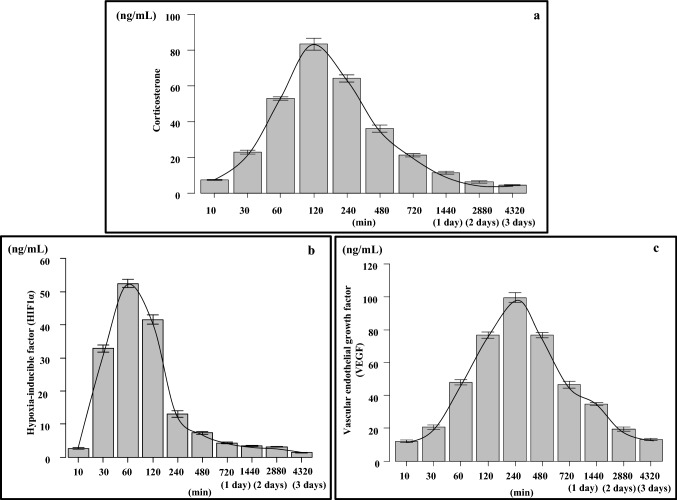
Corticosterone (**a**), HIF1α (**b**), and VEGF (**c**) concentrations in the conditioned medium Y-1 cultured under hypoxic conditions (O_2_ 1%). Corticosterone concentration levels were highest after 120 min of incubation under hypoxic conditions. HIF1α concentration levels were highest after 60 min, while the VEGF concentration levels were highest after 240 min of incubation under hypoxic conditions

The corticosterone level continued to increase from 10 min (7.1–7.9 ng/mL, median: 7.4 ng/mL) until it peaked at 120 min (79.7–88.6 ng/mL, median 82.6 ng/mL) during hypoxic conditions (Fig. 14a and Table 6). Levels of HIF1α and VEGF were used as control markers for cellular function during hypoxic conditions in Y-1 cells. HIF1α increased from 10 min (2.2–3.1 ng/mL, median 2.6 ng/mL) until it peaked at 60 min (50.6–54.4 ng/mL, median 52.8 ng/mL) (Fig. 14b and Table 6), and VEGF increased from 10 min (10.8–12.8 ng/mL, median 12.3 ng/mL) until it peaked at 240 min (96.7–105.2 ng/mL, median 98.5 ng/mL) (Fig. 14c and Table 6).

**Table 6:** Adrenal cortex cell (Y-1) culture under hypoxia

Cultured time under hypoxia (min)	Adrenal cortex cell culture (Y-1)		
	Corticosterone (ng/mL) (median)	HIF1α (ng/mL) (median)	VEGF (ng/mL) (median)
0	–	–	–
5	–	–	–
10	7.1–7.9 (7.4)	2.2–3.1 (2.6)	10.8–12.8 (12.3)
15	–	–	–
30	21.5–24.6 (22.8)	31.6–34.4 (32.6)	18.5–22.7 (20.6)
60	51.6–54.8 (52.6)	50.6–54.4 (52.8)	45.2–49.5 (48.3)
120	79.7–88.6 (82.6)	39.4–43.6 (41.8)	74.2–79.6 (76.2)
240	60.7–66.7 (64.3)	11.8–14.3 (12.8)	96.7–105.2 (98.5)
480	33.5–38.4 (35.5)	6.8–8.1 (7.2)	74.6–78.7 (77.1)
720	19.7–22.4 (21.3)	3.9–4.6 (4.2)	44.2–48 (46.4)
1440	10.3–12.6 (11.2)	3.2–3.6 (3.5)	33.7–36.3 (34.4)
2880	5.1–7.1 (6.6)	3.0–3.2 (3.2)	17.8–21.2 (19.4)
4320	4.1–5.0 (4.3)	1.2–1.5 (1.3)	12.3–14.1 (13.5)

## Discussion

We used three methods to evaluate pancreatic morphological changes under systemic hypoxia conditions. First, we examined the ratio and degree of the macroscopic subcapsular/interstitial hemorrhage. Second, we evaluated the pattern of the changes in the histologic tissue. Third, we investigated the insulin and glucagon-positive ratio in the islet cells as it related to each cause of death. Results showed that subcapsular/interstitial hemorrhage was observed as a macroscopic change in the pancreas after death from systemic hypoxia, which is commonly known as asphyxia. Hypoxic conditions have been shown to cause injuries to organ tissues and the vascular system [10, 44]. Eguchi et al. showed that the human blood vessel endothelium in vitro was cleared with the aid of Heat shock protein (Hsp) [45]. One of the common functions of Hsp is the inhibition of apoptosis. Thus, during the subcapsular/interstitial hemorrhage of the pancreas, the release of Hsp during hypoxia could potentially influence the changes that occur. It has also been reported that various structural proteins are damaged during conditions of hypoxia and needs to be taken into consideration [42, 46]. Additional work needs to be done to the mechanism of subcapsular/interstitial hemorrhage of the pancreas.

Based on these findings, it is highly likely that the pancreatic subcapsular/interstitial hemorrhage that was observed in the present study was the consequence of the hypoxia influence. Severe hemorrhage was more frequently observed in cases of death from asphyxia than in cases of death due to drowning. The pancreatic subcapsular/interstitial hemorrhage findings observed in cases of death due to drowning suggest that drowning involves systemic hypoxia. Other factors, such as alveolar injury, have also been reported to be integrally involved in the process of death [47, 48]. Pancreatic hemorrhage has been observed in approximately 40% of acute cardiac deaths. This finding may be attributable to systemic hypoxic injuries that result from circulatory failure due to cardiovascular injury [6]. In our pathohistological examination, tissue changes were frequently observed in asphyxia and acute cardiac deaths, and severe tissue changes were particularly observed more frequently in asphyxia. Immunostaining of the islets of Langerhans in the pancreas demonstrated that there was no difference in the percentages of the insulin- and glucagon-positive cells. The blood biochemistry tests revealed glucagon levels did not differ between the various causes of death, and the blood insulin levels in the asphyxia group were higher than those observed in all other groups. While these results suggest that hypoxic conditions can cause injuries to blood capillaries in the pancreas, insulin leakage does not appear to be attributable to cell disintegration. Pancreatic hemorrhage or micromorphological changes were not correlated with the blood insulin levels. In contrast, no differences were observed between the causes of death in relation to the secretion of pancreatic amylase from the pancreatic exocrine gland. All pancreatic amylase levels were within the clinical standard range. These results revealed that the endocrine gland was stimulated though the external secretion organization of the pancreas and was not affected by the acute hypoxia condition.

Previous research found that the mechanism underlying the increased blood insulin levels during hypoxic conditions could be explained by hyperglycemia induced by elevated cortisol levels [49]. Our previous studies have shown that asphyxia, a hypoxic condition does not induce any noticeable response in the blood cortisol level. We did find that exposure to cold induces a response in blood cortisol levels [50]. In this study blood cortisol levels did not differ markedly between the different causes of death.

We found blood glucose levels to be varied, but our results showed that there was no difference in the blood glucose levels observed in the deaths due to different causes. No correlation between blood glucose or cortisol levels and insulin levels was found. These results suggest that there is a direct effect of hypoxia on insulin secretion.

Based on these previous findings, we performed an experiment using Langerhans cells from the rat pancreas to observe changes in insulin mRNA and protein levels related to insulin secretion during hypoxic conditions. We found that insulin mRNA reached its peak in approximately 10 min during hypoxic conditions. Insulin protein levels also peaked around 10 min under hypoxic conditions and then decreased. Another research study reported that insulin increased after 2–3 min of hypoxia [49], which suggests that the pancreatic tissue is sensitive to acute conditions of hypoxia.

In rat islets the conversion of proinsulin to insulin begins about 30 min after ribosomal synthesis of preproinsulin and resembles first-order reaction kinetics with half-times of approximately 30–60 min [51]. However, the present cell culture experimental data, confirmed there were increases of both Ins1 mRNA and insulin during the first 10 min of hypoxia exposure. Furthermore, we found that the addition of actinomycin D starting at 24 h after the initiation of the culture led to no further changes in the insulin secretion quantity at each of the time points. These results suggest that the insulin secretion system is influenced by conditions of hypoxia. As the addition of actinomycin D in the cultured cells during conditions of hypoxia resulted in small changes in the HIF1α and VEFG.

The results of the hypoxia experiment with the pancreatic endocrine gland using BRIN-BD11 demonstrated that the HIF1α and VEGF increases occurred after the time insulin secretion increase was noted during hypoxic conditions. This suggests that the secretion of insulin during conditions of hypoxia was not affected by HIF1α.

Morphological changes in cultured pancreatic internal secretion cells during hypoxia were not noticeable for up to 10 min. Mitochondrial swelling and disintegration of nuclear structure only becoming noticeable at 15 min. Results of the electron microscopic examination also revealed that the activity of insulin secretion genes was not altered until around 10 min during hypoxic conditions. In contrast, there were no remarkable morphological changes in the organelles of the external secretion cell line (AR42J) as compared to the internal secretion cell line (BRIN-BD11) during conditions of hypoxia. No significant differences were observed during hypoxia for either the mRNA or amylase protein levels in the cultured cells. Hormone secretion and organelle changes during hypoxia were more pronounced in the pancreatic endocrine gland than the exocrine gland. Overall, these findings suggest that the insulin changes noted during conditions of hypoxia were due to a special reaction of the pancreatic endocrine system.

In autopsy cases, it is important that blood is collected from the vascular supply area central to the pathology [52]. This suggests that the portal vein would be the preferred blood collection site for studies of the pancreas. We opted to use blood collected from the right heart chamber because there is an insufficient amount of available via the portal vein. Our previous studies on amylase related to the pancreas were also conducted using blood predominantly collected from the right heart chambers [10].

Pancreatic cells cultured in 5% O_2_ were also used as a model of a hypoxic environment. The effects on hormone secretion were not clearly observed in the adrenocortical cells that were cultured under these conditions. When adrenocortical cells were cultured under a 1% O_2_ hypoxic environment, the corticosterone level reached its peak at 120 min. However, this finding does not support that insulin secretion under hypoxic conditions is clearly affected by corticosterone secretion. An independent experiment that we conducted to examine pancreatic cells under hypoxia, showed that hypoxia itself stimulated Langerhans cells in the pancreas to induce secrete insulin. The results from our present study demonstrate that increases of insulin related to the hypoxia stress are not dependent on either the cortisol or glucose levels.

## Conclusion

Current experimental results suggest that acute systemic hypoxic conditions can directly affect the mechanisms involved in pancreatic insulin secretion.
